# Residual Sterile Muscle Fibrosis Following Partial Debridement of a Pressure Ulcer

**DOI:** 10.7759/cureus.91989

**Published:** 2025-09-10

**Authors:** Joran Tilkin, Jos Velleman, Katarina Segers, Michel Van Brussel

**Affiliations:** 1 Plastic and Reconstructive Surgery, Universitair Ziekenhuis Leuven, Leuven, BEL

**Keywords:** advanced pressure ulcer, chronic wound management, pressure ulcer, pressure ulcer management, radical debridement

## Abstract

Pressure ulcers are severe medical conditions, often stemming from inadequate pressure redistribution. Proper wound management is vital to ensuring optimal healing outcomes. Surgical intervention is often indicated and consists of the removal of devitalized tissue down to viable tissue in order to facilitate optimal wound healing, either via surgical reconstruction or per secundam. A 52-year-old man with paraplegia and recurrent respiratory infections developed a pressure ulcer on his right upper leg. Under conservative measures, the ulcer persisted, accompanied by a prolonged fever. Surgical debridement was performed down to the muscle fascia, intentionally preserving the covered avital muscle for subsequent sterile fibrosis. Pressure ulcers present substantial clinical challenges, particularly in patients with extensive comorbidities. In such cases, traditional surgical management may not always be the most feasible or optimal approach. Partial debridement, with a focus on preserving the muscle fascia, provides an effective means for removing infected tissue while allowing the remaining tissue bulk to undergo sterile necrosis and fibrosis. This approach serves as an interim strategy in complex pressure ulcer cases, avoiding significant muscle loss and thus circumventing the necessity for reconstructive flap surgery for sizable wound defects. Complex pressure ulcers in high-comorbidity patients present significant challenges in wound management. The concept of partial debridement with remaining sterile muscle fibrosis can serve as an interim solution, facilitating conservative wound healing.

## Introduction

Pressure ulcers, also known as bedsores or pressure sores, are a type of injury that occurs when prolonged pressure is applied to a particular area of the skin, resulting in tissue breakdown, ischemia, cessation of nutrition and oxygen supply to the tissues, and eventually tissue fibrosis [1]. Pressure ulcers are serious medical conditions, so prevention is crucial, with pressure redistribution as the main focus. This includes frequent body repositioning, a low bed incline angle, and optimal patient positioning. Additionally, reducing pressure magnitude is essential, and various support surfaces, such as specialised beds, mattresses, overlays, and cushions, are available to achieve this goal [2].

Today, the treatment of pressure ulcers is based on pressure reduction, prevention of additional ulcers, wound management, surgical intervention, mobilization, and improvement of the general condition, often with an emphasis on nutrition. Early stages prefer conservative measures, with treatment options, including wound dressings, antiseptics, and occasionally antibiotics [3]. In this stage, wound management is critical to ensuring effective healing. The key aspects are the cleaning of the wound, effective drainage, and absorption of wound moisture while protecting the skin adjacent to the wound [4].

Surgery is indicated when the removal of devitalized tissue is required to optimize wound healing. The removal of moist necrotic tissue is essential to avoid a medium for infection that triggers an inflammatory response and can eventually lead to sepsis. Small or superficial wounds can be debrided at the bedside, but debridement in the operating room is often required for advanced pressure ulcers [5]. Several surgical techniques exist to heal the wound, ranging from surgical debridement to skin grafts, flap surgery, or a combination of these. The method used by the surgeon depends on the severity of the wound and the physical status of the patient [3]. We introduce a novel concept, “sterile fibrosis,” as an alternative pathway to healing, particularly suited for patients with complex comorbidities for whom standard surgical methods are too invasive. It is essential that any debridement aligns with a structured treatment plan, carefully outlining each step needed for progressive and safe healing. This approach is demonstrated through a case discussion.

## Case presentation

This case report was approved by the Research Ethics Committee of the University Hospitals Leuven, and informed consent was obtained according to the local guidelines (S69211).

On 4 March 2024, a 52-year-old man was admitted to the hospital, presenting with dyspnoea and fever. He was later diagnosed with bilateral segmental and subsegmental pulmonary emboli. He had a known closed pressure ulcer on his right upper leg, initially diagnosed in January 2024 during his ICU admission for a severe chronic obstructive pulmonary disease (COPD) exacerbation. His current daily medication included amlodipine 5 mg, mirtazapine 45 mg, quetiapine 150 mg, and trazodone 50 mg. The patient had a medical history of paraplegia after back surgery in 2004 because of persistent dorsalgia from a T7 fracture, multiple pressure wounds (heel, knee, foot, hip), neurogenic bladder with a suprapubic catheter, meningitis due to an intrathecal baclofen pump, obesity, COPD Global Initiative for Obstructive Lung Disease (GOLD) IIIE, alpha-1 antitrypsin deficiency, and anxiety and sleep problems.

**Figure 1:**
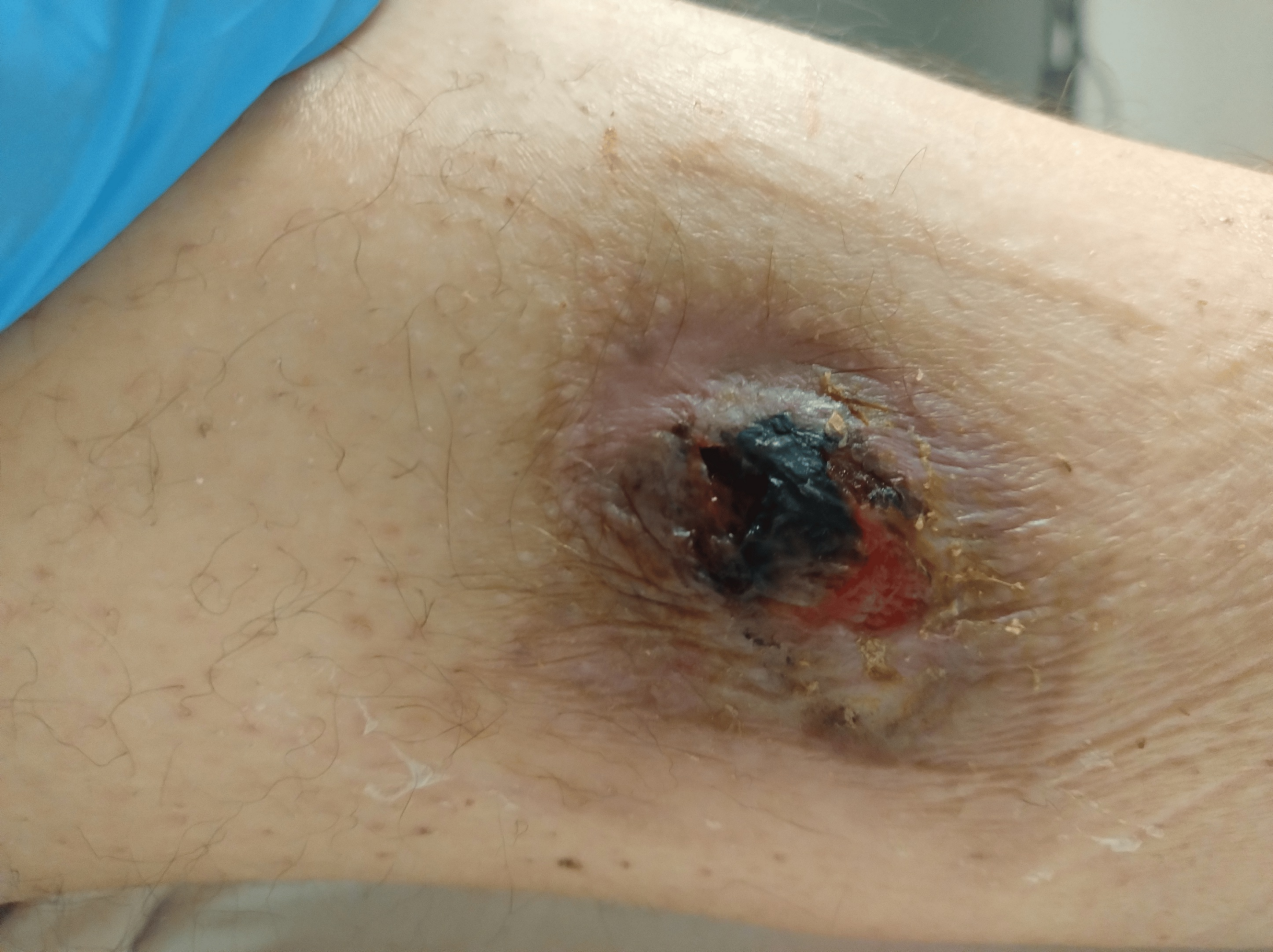
Initial wound presentation in January, treated with Flaminal Forte®.

**Figure 2:**
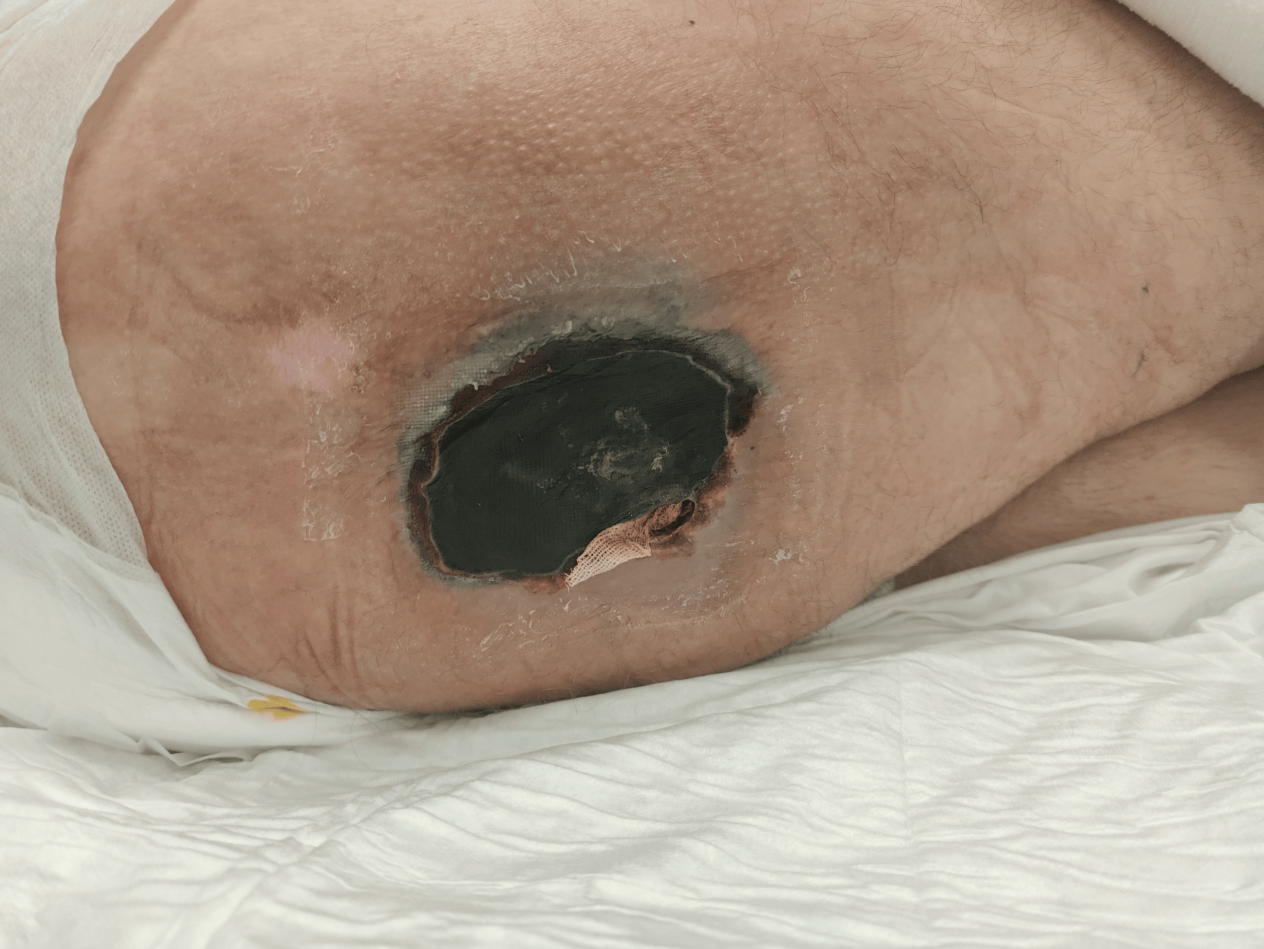
During the hospitalization in March, a dry necrotic crust of approximately 8 cm was noted (dated 15/03/2024). A small opening on the caudal posterior side was observed, accompanied by minimal drainage. While some redness was observed at the wound margins, there were no indications of underlying infection or abscess formation. Consequently, the wound was addressed using povidone-iodine tulle and a wick. However, after several days, an aperture in the crust appeared, accompanied by the discharge of pus with an unpleasant odour.

The pressure ulcer on the posterior-lateral side of the right upper leg was likely caused by friction or pressure from an improperly adjusted wheelchair. The initial wound care plan in January involved cleaning with physiological 0.9% saline, disinfecting with povidone-iodine solution, and application of Flaminal Forte® (Flen Pharma, Kontich, Belgium) (see Figure 1). Upon hospitalization in March, the wound care regimen was changed to povidone-iodine tulle and impregnated wick (see Figure 2). Following the recent admission and subsequent pus culture, which revealed *Klebsiella pneumoniae* and *Enterococcus faecalis*, treatment with piperacillin/tazobactam was initiated. The patient was instructed to diligently adhere to decubitus prevention measures, and a more suitable wheelchair was provided.

**Figure 3:**
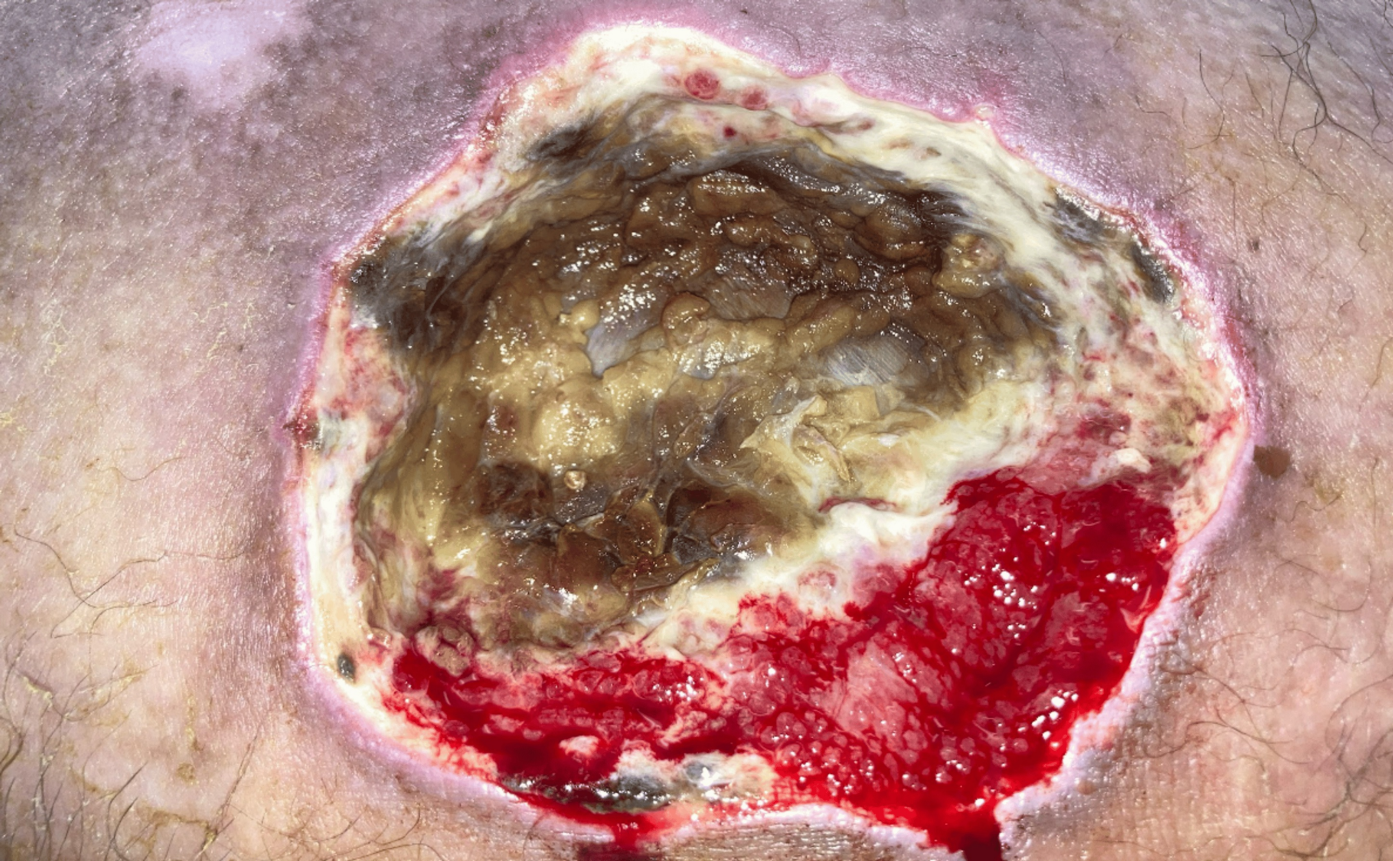
Following the second bedside wound debridement, vital granulation tissue was observed alongside central necrotic-smelling subcutaneous tissue. This image depicts the status prior to surgical debridement (dated 25/03/2024).

On the 21st of March, there was a noticeable lateral extension of the dry necrotizing crust of the wound, accompanied by an enlarged opening and the discharge of malodorous pus. Given the patient’s persistent fever, with the pressure ulcer as the sole focus, bedside debridement was performed to remove the necrotizing crust. This procedure resulted in the removal of a significant amount of necrotic soft tissue, revealing minimal granulation at the wound base. Subsequently, wound care was transitioned to povidone-iodine gel smeared compresses. In response to the bacterial resistance profile, piperacillin/tazobactam was replaced with amoxicillin and co-trimoxazole. On 23rd of March, a second bedside debridement was carried out to eliminate the remaining infected tissue deeper down to the subcutis, eliciting minor haemorrhage, which was managed with a tranexamic acid compress (see Figure 3).

**Figure 4:**
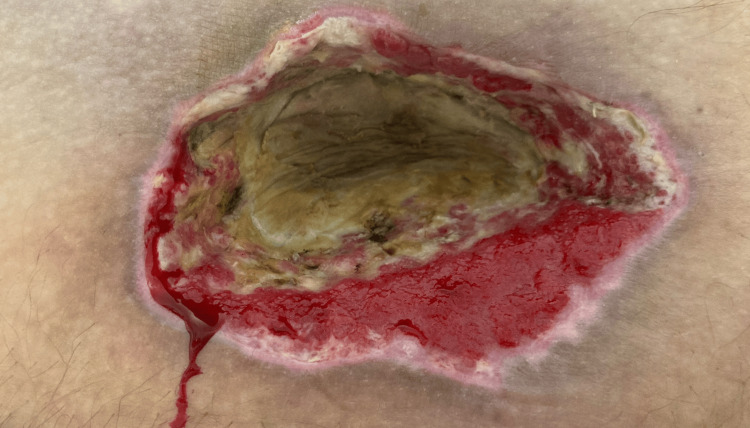
Post-surgical debridement, vital granulating tissue was evident around the wound edges, while the central area displayed dry necrotic tissue (dated 09/04/2024).

On the 25th of March, the patient continued to suffer from a fever of 38.6°C despite antibiotic treatment, with the wound remaining the only possible source. There was deterioration of the wound, prompting a surgical debridement on the 4th of April, as the previous bedside debridement was deemed insufficient. Prior to the surgical procedure, edoxaban was started during hospitalization for a lung embolism and was used with enoxaparin. The surgical intervention involved excising the necrotizing wound edges and bottom, accompanied by thorough curettage and irrigation with povidone-iodine. The muscle fascia was preserved to keep the muscle tissue covered with the aim of protecting it from infection and thus inducing sterile necrosis, followed by subsequent fibrosis (see Figure 4). Wound cultures revealed the presence of *Staphylococcus epidermis* and *Enterococcus avium*. The post-operative course was complicated by respiratory failure and difficult weaning.

**Figure 5:**
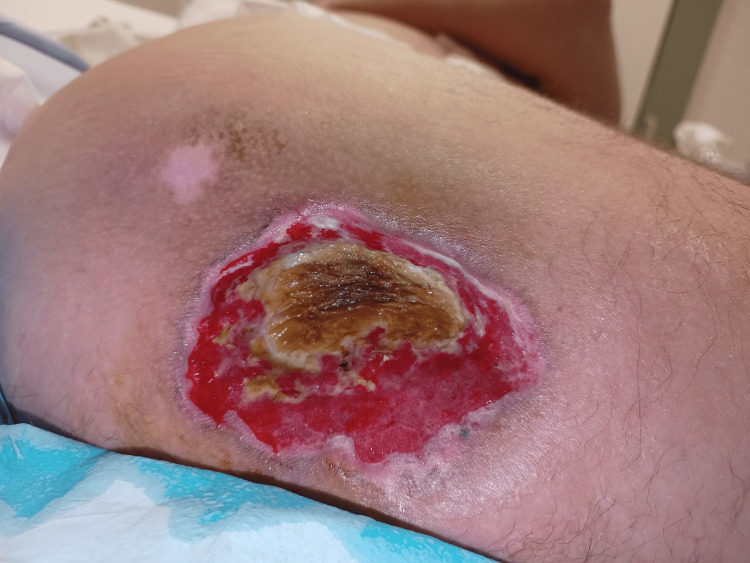
Sixteen days after surgery, clinical improvement was noted with larger vital granulation tissue around the wound edges and contracture of the fibrotic tissue (dated 20/04/2024).

After surgical debridement, daily wound care included the application of povidone-iodine. To prevent pressure on the wound, the patient was required to lie on his left side. On 5 April, antibiotic therapy was transitioned to vancomycin following the detection of four positive haemocultures for *Staphylococcus hominis*. The patient's last episode of fever was noted on 8 April, after which mobilization efforts commenced. Subsequently, the wound appeared clean with no signs of infection. Clinically, there was evident improvement in the wound condition, characterized by the presence of more vital tissue (see Figure 5).

There was further clinical improvement of the wound, with increased contraction of the central sterile fibrosis and a reduction in wound size (dated 21/05/2024).

**Figure 6:**
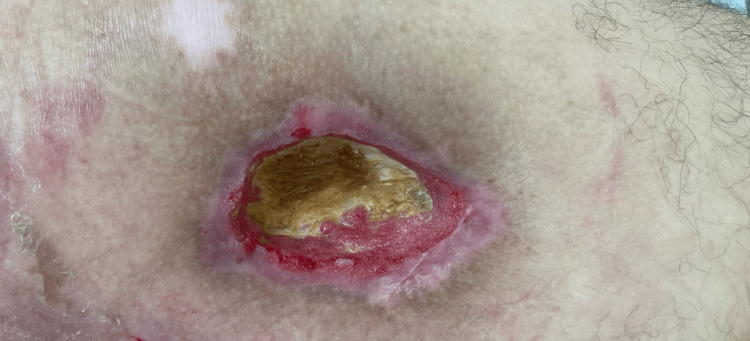
There was further clinical improvement of the wound, with increased contraction of the central sterile fibrosis and a reduction in wound size (dated 21/05/2024).

The patient's fever resolved following surgery. On the 15th of April, negative pressure wound therapy was initiated once sufficient improvement was observed without signs of infection. This therapy was briefly paused due to a short episode of fever. On the 26th of April, closed system negative pressure irrigation was started, and clinical improvement of the wound was observed (see Figure 6).

Unfortunately, the patient's respiratory status deteriorated. After discussions with the patient and his family, a collective decision was made to provide comfort therapy. The patient passed away on the 27th of May due to respiratory failure from COPD GOLD III-E.

## Discussion

Surgical management of pressure ulcers is typically considered for Stage 3 or Stage 4 ulcers exhibiting necrotic tissue and failing to heal despite conservative measures. Additionally, surgical intervention becomes necessary when osteomyelitis or sepsis is suspected. While small ulcers may sometimes allow for primary closure, this approach is seldom utilized. Skin grafts are an option for superficial wounds, but for Stage 3 or Stage 4 ulcers, tissue flap closure is often required. The surgical team may opt for local, regional, or distant/free flaps based on ulcer size and location [1].

In the case described, an unconventional approach was taken to address the pressure ulcer. Given the patient's fever and systemic symptoms, conservative non-surgical treatment proved insufficient. However, the patient's poor overall condition posed a high risk of flap failure and postoperative complications. Consequently, a sharp surgical debridement was performed, removing infected tissue down to the muscle fascia. Intentionally retaining necrotic muscle beneath the fascia, the objective consisted of sterile necrosis and subsequent fibrosis of the muscle. The final management plan entailed conservative wound healing through contracture of the fibrotic tissue and gradual coverage of granulation from the surroundings.

This approach offered the advantage of avoiding flap surgery in a patient deemed unsuitable for such a procedure. Studies indicate that patients with paralysis and significant comorbidities face increased risks of flap failure, with a recurrence rate of 27% over a two-year period [6,7]. Primary closure or skin grafting was not feasible due to the extent of the pressure ulcer. However, the incomplete debridement, deviating from standard recommendations, poses a significant risk. Retaining vital tissue carries a high risk of infection, resulting in the failure of this partial debridement. Therefore, this technique is only suitable for specific cases involving complex pressure ulcers, where the advantages and disadvantages of potential surgical interventions must be carefully weighed. Moreover, this unique approach can only be applied when the underlying muscle doesn’t show any direct signs of infection; if so, there is a strict indication to remove the infected muscle.

The concept of sterile fibrosis, which can occur in compartment syndrome but is not described in the literature in the context of pressure sores, was applied here as an interim measure for a patient who failed conservative treatment but was not a candidate for standard reconstructive surgery. Since the patient suffered from paraplegia, muscular function was of lesser importance. We considered that opening and removing the fascia would pose a major risk of infection of the muscle. This, in turn, would have led to additional debridements with the removal of the muscle and a need for even larger reconstructions in a patient in poor general condition. Inevitably, this would have led to disarticulation, with a resulting corporeal imbalance. Sparing the muscle was a calculated risk insofar as, when successful, it would have led to less invasive treatment and a better quality of life.

## Conclusions

Partial debridement of a pressure ulcer until the muscle fascia, with the preservation of sterile muscle fibrosis, represents a possible approach for managing pressure ulcers in patients with a high-risk profile still necessitating surgery. This case suggests an alternative possibility in pressure ulcer management, underscoring its potential efficacy.
